# Evaluation of multidisciplinary high-risk pregnancy clinic for myelomeningocele

**DOI:** 10.1007/s00381-024-06337-4

**Published:** 2024-04-22

**Authors:** Luke Anderson, Betsy Hopson, Caroline Caudill, Brandon G. Rocque, Jeffrey Blount, Anastasia Arynchyna-Smith, Jessica Thrower, James Johnston, Curtis Rozzelle

**Affiliations:** 1 Marnix E. Heersink School of Medicine, University of Alabama at Birmingham, Birmingham, AL USA; 2 Department of Medicine, University of Alabama at Birmingham, Birmingham, AL USA; 3 Department of Neurosurgery, University of Alabama at Birmingham, Birmingham, AL USA; 4 Division of Pediatric Neurosurgery, University of Alabama at Birmingham, 1600 7th Ave S., Lowder 400, Birmingham, AL USA

**Keywords:** Spina bifida, Myelomeningocele, Prenatal clinic, Lifespan care, Prenatal counseling

## Abstract

**Introduction:**

A cross-sectional study retrospectively evaluating the perceived usefulness of attending a multi-disciplinary, roundtable, educational prenatal clinic for mothers expecting children with myelomeningocele is presented.

**Methods:**

Mothers who currently have children with SB completed a survey which evaluated their overall preparedness, spina bifida education, delivery plans, surgical expectations, and expectations in terms of quality of life and development. Open comments were also collected. Statistical analysis was performed to identify differences between those who attended prenatal counseling and those who did not.

**Results:**

Approximately half of these mothers received some form of prenatal SB counseling. Mothers who attended prenatal counseling reported that they felt more informed and prepared throughout their pregnancy, during the delivery of their child and during their initial hospital stay than mothers who did not. They reported that the roundtable discussions were beneficial, and the education they received was useful in helping them form accurate expectations and feel more at ease.

**Conclusion:**

This suggests that prenatal counseling and the High-Risk Pregnancy Clinic (HRPC) provides perceived utility to families and mothers and that the HRPC is an effective method of providing prenatal counseling to mothers whose unborn children have been diagnosed with myelomeningocele.

## Introduction

Myelomeningocele, the most common neural tube defect, involves the incomplete fusion of the posterior spinal column, leading to the protrusion of neural tissue and meninges through the fetus’s posterior aspect [1]. This condition lacks skin coverage; thus, maternal tests reveal elevated alpha-fetoprotein (AFP), and ultrasound typically confirms the prenatal diagnosis.

Despite routine multidisciplinary care for myelomeningocele at the authors’ institution post-birth, many mothers experience significant anxiety and stress during pregnancy before such care is established for their child.

Recognizing that fear and anxiety are often exacerbated by the unknown, it was hypothesized that enhanced education and communication could improve the experience of mothers and families during pregnancy and childbirth. The High-Risk Pregnancy Clinic (HRPC) at Children’s of Alabama (COA) operates within the Lifetime Care Model, aiming to transition from solely providing care through a spina bifida (SB) clinic to establishing a longitudinal program with ongoing communication across specialties [2].

The HRPC, a maternal–fetal clinic, employs a multidisciplinary approach to prenatal family education, involving pediatric specialists, surgeons, a high-risk OB-GYN physician, and an SB Coordinator. Its goal is to educate families, aid in decision-making, and address their concerns. Topics covered include quality of life expectations, potential complications, disease process, and surgical decisions, such as hydrocephalus management. The clinic also addresses crucial decisions like mode of delivery and timing of closure. Families are provided with an SB Handbook and introduced to COA’s multidisciplinary SB clinic, ensuring seamless care coordination within the Lifetime Care Model. The HRPC is unique in offering education regarding all levels of SB care including discussions about in utero closure of the myelomeningocele although our center does not currently offer fetal surgery [3, 4]. However, due to the timing and logistics of HRPC, many families have already decided on pursuing post-natal closure with our center by the time of their initial HRPC visit.

The Lifetime Care Model at COA was developed through action-research-reflection cycles, evolving to meet the needs of individuals affected by SB in Alabama [5]. The HRPC, as the model’s initial stage, is an evidence-based program continually evaluated for effectiveness [2].

This study aims to assess the perceived usefulness of the HRPC, determine its value to patients, and identify areas for improvement. Specifically, it seeks to evaluate whether the clinic enhances families’ preparedness during pregnancy and which aspects are most effective.

## Materials and methods

The study was a prospective, cross-sectional survey conducted among birth mothers whose children have myelomeningocele and are being followed in the multidisciplinary SB clinic at COA, which occurs bi-monthly.

This clinic caters to families with children affected by various forms of spinal dysraphism, including myelomeningocele, tethered cord, sacral agenesis, caudal regression, and fatty filum. Given the prevalence of myelomeningocele as the most commonly diagnosed prenatal spinal dysraphism, the study focused on families with children specifically diagnosed with myelomeningocele. As the HRPC at UAB had been established approximately 10 years before the project’s inception, the study included only mothers of children aged 10 or younger. Enrollment was limited to English-speaking mothers due to the absence of a Spanish survey. Additionally, as the HRPC involved the mother during pregnancy, only families with biological children affected by myelomeningocele were included.

Patient lists were screened prior to clinic days to identify potential participants based on the diagnosis of myelomeningocele and the child’s age. On clinic days, researchers confirmed the presence of the biological mother and her English fluency. Following informed consent, the mother was provided with an iPad to complete demographic information and the subsequent survey. To ensure privacy and encourage honest feedback, researchers left the room while mothers completed the survey. Responses were coded with a unique participant ID, and no identifying information was requested on the survey.

After providing demographic information, mothers indicated whether they had received prenatal counseling. Depending on whether prenatal counseling had occurred (either through the HRPC or elsewhere), mothers completed slightly different surveys. If the mother attended a similar program at another institution, the institution was noted. The survey comprised pre-written statements (available in Appendix 3) to which mothers responded using a five-point Likert scale (1 = strongly disagree; 5 = strongly agree). All participants responded to 8 statements, with an additional 16 statements for mothers who attended some form of HRPC, and 2 additional statements for mothers who did not attend any HRPC. Mothers evaluated their overall preparedness throughout the perinatal process, the SB education received, thoughts on delivery plans and surgical expectations, and expectations regarding their child’s quality of life and development. Open comments were also collected to identify the most useful aspects of the educational process, any information that induced anxiety, and suggestions for additional education desired by mothers and families.

Study data were collected and managed using REDCap electronic data capture tools hosted at UAB [6, 7]. Survey responses, utilizing a five-point Likert scale, were coded numerically to facilitate analysis. This cross-sectional study aimed to survey all eligible mothers whose children received routine care during a 2022 multidisciplinary SB clinic visit at COA.

For statements posed to both groups, chi-squared tests were conducted to analyze differences in response rates between those who attended prenatal counseling and those who did not. Descriptive statistics are reported for additional questions exclusively answered by HRPC attendees or those who did not receive prenatal counseling. No statistically significant differences were found between the responses of mothers who attended HRPC at UAB and those attending similar clinics elsewhere. Hence, all mothers who attended prenatal counseling were grouped together for statistical purposes. Additionally, an independent sample *T*-test was conducted to compare the number of surgeries undergone by children.

## Results

**Chart 1:**
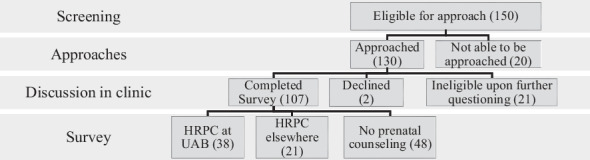
Screening Process Flow Chart

The following data are summarized in Chart 1: During the 10 months the survey was distributed, 150 families met the screening criteria. Of these, 20 were not approached in clinic (11 did not show up to their appointment, 9 departed before engagement with the research team). During initial discussions, 21 families were ineligible, primarily because the biological mother not being involved with the regular healthcare of the child (mainly due to the large population of adopted patients). Additionally, some families were ineligible due to language barriers. Of the 109 eligible families, 2 declined due to personal reasons, leaving 107 families to complete the survey. Among these respondents, 48 did not receive prenatal counseling, 38 received counseling at UAB, and 21 received counseling at a different institution’s clinic. Notably, only families referred to UAB high-risk OB during pregnancy were referred for the HRPC.

The demographics of the participants are shown in Table 1.

**Table 1:** Demographics of the participants

**Race/ethnicity**	Number of respondents (% of sample)	
White, non-Hispanic	64 (59.8%)	
Black	29 (27.1%)	
Hispanic	7 (6.5%)	
Other	7 (6.5%)	

**Educational attainment**	**Number of respondents (% of sample)**	
	**At time of child birth**	**At time of completion of survey**
Some high school (HS)	4 (3.7)	2 (1.9)
HS graduate/GED	33 (30.8)	24 (22.6)
Some college/TS	4 (3.7)	5 (4.7)
TS graduate	26 (24.3)	30 (28.3)
College graduate	31 (29.0)	35 (33.0)
MS degree	7 (6.5)	8 (7.5)
Professional degree	2 (1.9)	2 (1.9)

**Household income**	**Number of respondents (% of sample)**	
	**At time of child birth**	**At time of completion of survey**
$0– < $20,000	29 (27.4)	19 (17.9)
$20,000– < $40,000	26 (24.5)	20 (18.9)
$40,000– < $60,000	13 (12.3)	16 (15.1)
$60,000– < $80,000	8 (7.5)	16 (15.1)
$80,000– < $100,000	15 (14.2)	10 (9.4)
$100,000– < $150,000	8 (7.5)	15 (14.2)
$150,000 +	7 (6.6)	10 (9.4)

**Fig. 1:**
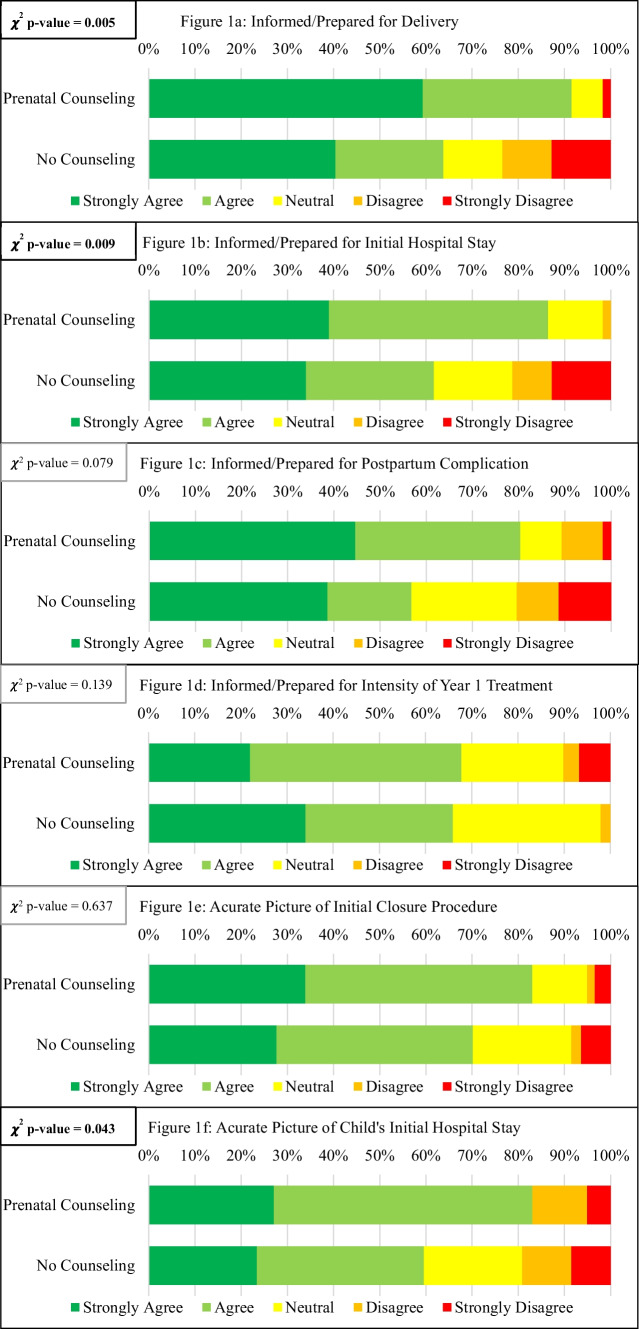
Participant responses to statements about how informed/prepared they felt for the delivery of their child (**a**), their initial hospital stay (**b**), postpartum complications (**c**), and the intensity of year 1 treatment (**d**) and how accurate they felt their expectation of their initial closure procedure (**e**) and their initial hospital stay (**f**) were to their lived experience, the chi-squared analysis comparing mothers who attended prenatal counseling to those who did not

Figure 1 shows responses to a series of statements that all participants responded to, regardless of their prenatal counseling experience. The proportions of responses from mothers who attended prenatal counseling were directly compared to those who did not, using chi-squared tests.

Figure 1a and b reveal a statistically significant difference in how well informed and prepared mothers felt about the delivery of their child and their initial hospital stay. Mothers who attended prenatal counseling reported feeling more informed and prepared. Similarly, Fig. 1f demonstrates a statistically significant difference in how accurate mothers felt their expectations were about the initial hospital stay, with those who attended prenatal counseling feeling more informed and prepared.

The average number of surgical procedures that the child underwent during their first year of life did not show a statistically significant difference between the two groups when compared using an independent sample *T*-test (*p*-value of 0.823). Children of mothers who attended prenatal counseling had an average of 3.800 surgeries in the first year of life whereas children of mothers who did not attend any prenatal counseling had an average of 3.619. The mean difference between these two groups was 0.181 surgeries with a 95% CI of − 1.460 to 1.822.

**Fig. 2:**
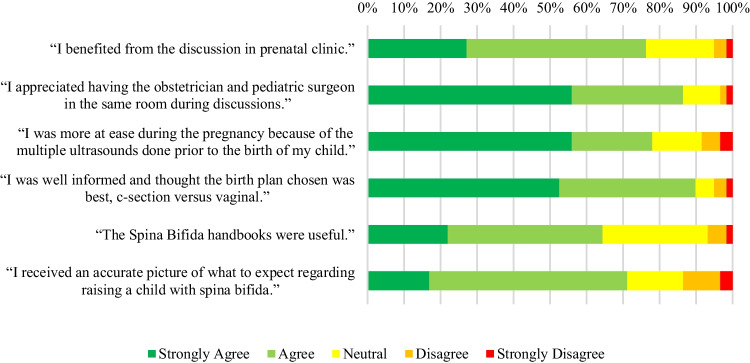
Participant responses to statements about how they felt about various elements of their prenatal counseling experience

**Fig. 3:**
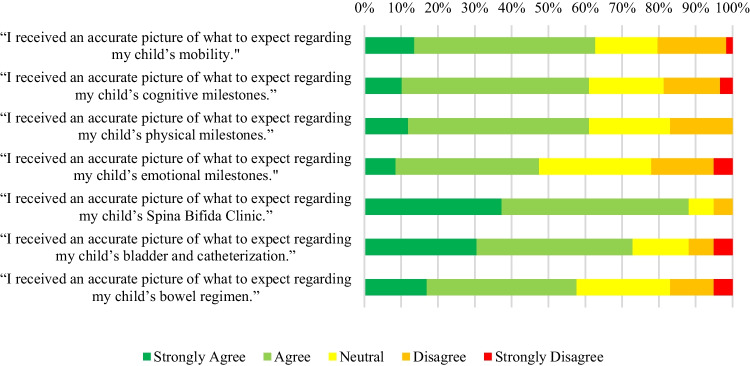
Participant responses to statements about how accurate they felt their expectations about their child were compared to their lived experience

**Fig. 4:**
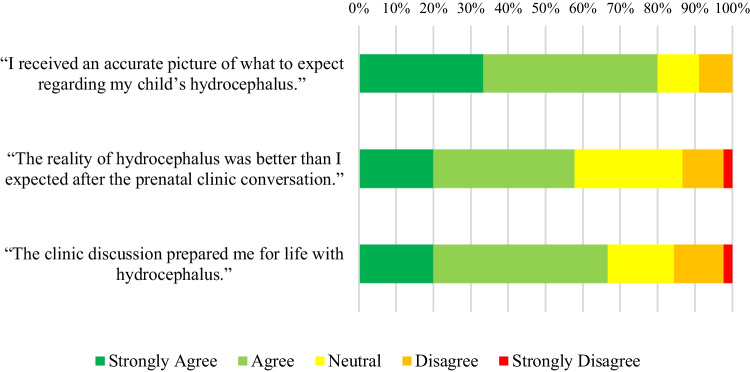
Responses of participants whose child also had a diagnosis of hydrocephalus to statements about their expectations and experiences of life with hydrocephalus

**Fig. 5:**
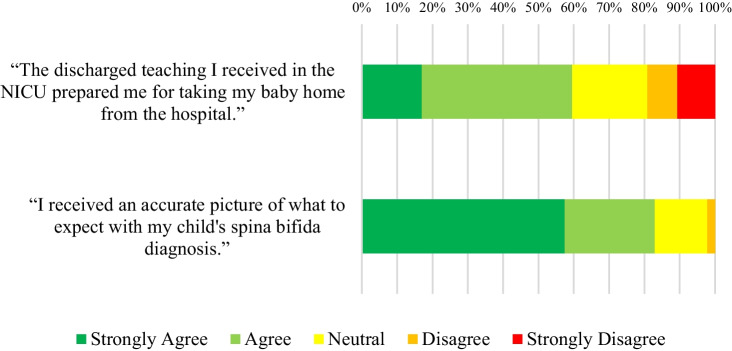
Responses of participants who did not go through any prenatal counseling to statements about their level of preparedness

Figures 2, 3, 4, and 5 show the responses of the prenatal counseling group to specific questions about their prenatal counseling experience.

Figure 2 illustrates that the preponderance of mothers responded positively to all six statements.

Mothers who attended prenatal counseling were asked to respond to a series of question to quantify how well their expectations during and after attending prenatal counseling matched their experienced reality. Their responses are displayed in Fig. 3.

Figure 3 illustrates that the preponderance of mothers felt that their expectations formed in prenatal counseling matched their lived reality.

Of the mothers who attended prenatal counseling (59), 45 had children with a concurrent diagnosis of hydrocephalus. This sub-group was asked to respond to three additional statements. Their responses are displayed in Fig. 4.

Figure 4 illustrates that the majority of mothers responded positively to all three statements.

Mothers who did not attend prenatal counseling were asked to respond to two additional statements, to evaluate their subjective preparedness. Their responses are depicted in Fig. 5.

Figure 5 illustrates that the majority of mothers responded positively to both statements.

## Discussion

The current literature increasingly documents the use, implementation, and effectiveness of multidisciplinary clinics akin to the HRPC [8–16]. Like the HRPC, these clinics serve as platforms for patient and family education, care planning, and discussions regarding crucial surgical and medical choices [8–15]. These clinics extend beyond the realm of SB, catering to individuals with conditions such as pancreatic cancer, breast cancer, pediatric single-ventricle heart disease, complex dyslipidemia, epilepsy, and diseases necessitating long-term tracheostomy. They have also been instrumental in enhancing care delivery in family medicine clinics [8–11, 13, 14, 16].

Through comparing the responses of mothers who received prenatal counseling with a similar group of mothers who did not undergo such counseling, the observed differences in responses are likely attributable to the prenatal intervention. This comparison method has been effective in evaluating the efficacy of other multidisciplinary clinics [9]. Mothers who participated in prenatal counseling reported feeling more informed and prepared for their child’s delivery and initial hospital stay compared to those who did not. They also noted more accurate expectations regarding their child’s initial hospital stay. The majority of mothers attending HRPC expressed benefiting from the educational handbooks provided during their visit and feeling more reassured during pregnancy due to the increased frequency of ultrasounds. These findings echo those of other clinics, highlighting the importance of receiving reliable information from the medical team, which significantly enhances patients’ experiences [10, 12, 14]. Mothers likely experienced greater ease with decision outcomes, given their involvement in the decision-making process, a major desire expressed by patients attending multidisciplinary clinics [10, 12–14].

Mothers who participated in prenatal counseling also noted that the expectations they formed during these discussions aligned with their actual experiences. These expectations predominantly pertained to raising a child with SB, encompassing factors such as their child’s mobility, cognitive and physical development, emotional milestones, and bowel and bladder care routines. Previous research indicates that the caregiving responsibilities for children with SB in their early years can be significant, underscoring the importance of establishing accurate healthcare expectations during this period to alleviate overall caregiver burden [17]. Moreover, when provided with more information, patients and their families report an enhanced quality of life [13].

The majority of mothers who participated in prenatal counseling found significant value in the roundtable discussions. They appreciated the presence of the obstetrician and pediatric neurosurgeon during these sessions, which covered topics related to prenatal and postnatal care. These discussions facilitated the creation of personalized care plans and recommendations, emphasizing patient-centered care, something desired and appreciated by patients [12–15]. Research indicates that enhanced coordination among the medical team not only boosts patient satisfaction [8–16] but also yields better clinical outcomes [8, 9, 13]. Furthermore, these discussions have been shown to expedite the time to definitive diagnosis and subsequent interventions, both educational and medical [9]. The perceived unity among the medical team increases patients’ trust in the medical process, contributing to a positive patient experience [11, 15]. Patient attendance at these roundtable discussions results in more effective communication and coordination [12, 14, 15], along with improved continuity and coherence of care [16]. Consolidating multiple providers into one session also reduces the burden of managing numerous appointments across various clinics, enhancing convenience for families [11].

The majority of mothers who participated in prenatal counseling whose children were later diagnosed with hydrocephalus felt adequately prepared for this possibility. Fortunately, the actual experience of managing their child’s hydrocephalus was often better than their initial expectations formed during prenatal counseling. They felt that prenatal counseling prepared them for parenting a child with hydrocephalus. This finding aligns with the understanding that access to more information and resources empowers parents and patients to manage care independently at home with greater confidence [14]. For instance, one parent mentioned that their increased awareness of warning signs for shunt failure resulted in reduced anxiety, as they knew when to seek appropriate medical attention promptly.

However, no significant differences emerged between the two groups in their responses to statements regarding their preparedness for post-birth complications and the anticipated level of treatment required in their child’s first year of life. Similarly, there was no difference in the accuracy of mothers’ expectations regarding the initial closure procedure. These findings prompt a critical discussion: “How much is too much?” While the HRPC’s objective is to alleviate anxiety and fear, clinicians must delicately balance providing reassurance and hope with maintaining a realistic perspective on potential future challenges.

There were no significant differences in the number of surgical procedures undergone by patients in their first year of life. This is natural and expected, as prenatal counseling never aimed to alter objective surgical outcomes but rather to help the mothers be prepared for such outcomes. However, other studies observing clinical outcomes have shown that participation in multidisciplinary clinics leads to improved clinical outcomes [8, 9, 13].

Despite not having the benefit of the prenatal counseling discussions, the majority of mothers who did not receive prenatal counseling responded positively when asked about their expectations and preparedness leaving the neonatal intensive care unit (NICU). This could be due to limitations in the study, mentioned below.

In their open-ended comments, mothers who participated in HRPC highlighted two significant benefits: firstly, the opportunity to meet the SB coordinator, who guided them through their intricate medical care both before and after birth, and secondly, the chance to connect with other families whose children have SB. These observations underscore the importance of care coordination, offering families a direct point of contact for the care they will receive and facilitating connections to social support groups. Previous studies have emphasized the critical significance of introducing long-term care coordination [8] and providing social support [13].

During the prenatal period, several options are available for maternal care. These include meeting with a high-risk obstetrician at a pediatric center, attending HRPC with roundtable discussions, or establishing visits with multiple providers separately. It is important to acknowledge that studies have demonstrated the importance of clinic convenience to patients and their families [10, 11, 15]. The advent of telemedicine has significantly expanded access to this type of care, which was previously restricted to academic institutions. Telemedicine is perceived as offering quality care equivalent to in-person visits [15]. Additionally, telemedicine enhances the ease and convenience of the process for both providers and patients, further reducing the burden on caregivers and families [15].

## Limitations

This study had several limitations inherent to its retrospective and recall bias nature. Mothers were not randomly assigned to receive prenatal counseling, introducing the possibility of unaccounted confounding variables influencing counseling allocation.

The survey was administered after mothers had lived with and cared for their child for varying durations, ranging from a few months to 10 years. Consequently, all mothers, irrespective of prenatal counseling attendance, may now feel prepared to navigate the challenges associated with SB. However, this may not accurately reflect their feelings upon leaving the hospital. Furthermore, the absence of a concrete reference for “feeling prepared” adds complexity to interpreting the results.

With only 38 surveyed mothers attending HRPC, they were grouped together with those attending similar prenatal counseling elsewhere for statistical purposes. This amalgamation reduces result specificity, underscoring the potential value of multidisciplinary educational prenatal clinics for myelomeningocele more broadly, not solely limited to the HRPC at UAB/COA.

## Conclusion

Prenatal counseling and the HRPC provides perceived utility to families and mothers. Mothers who attended HRPC felt more informed and prepared, with more accurate expectations than their counterparts who did not attend HRPC. The majority of mothers who attended HRPC found it to be a helpful and useful process. HRPC is an effective method of providing prenatal counseling to mothers whose children have been diagnosed with myelomeningocele.

## Data Availability

No datasets were generated or analysed during the current study.

## Appendix 1. Suggested comments to families during the prenatal time-period

1. “The best questions often come up on the drive home. In your packet, I have included a phone number to get back in touch with me directly when those questions arise.”
2. “Your SB coordinator will become a small part of your family. They will help be the glue that holds all the pieces together, including all your appointments, tests, imaging, and hospital/clinic information.”
3. “First, Congratulations on your new baby! It is right and natural to be excited and to celebrate your new baby. There will be a long list of things that define who your baby is and who they will become, and SB is only one thing on that list.” “Sometimes, I have headaches, but I am not a headache, I am a person with hopes, dreams and life. Your baby will have spina bifida. However, your baby is not ‘a spina bifida.’ He/she will also have hopes dreams and a life that are not defined by spina bifida.”
4. “Now that you are here, you have an entire team of people to rally around you to support you through this process. Do not be afraid to ask question or give us a call – that is what we are here for.”

## Appendix 2. Suggested advice to SB coordinators

1. Be willing to take the whole family on a tour of all the places they will soon become familiar with. This includes the NICU, the clinic spaces of the providers they will be seeing, where they will be going to get lab and bloodwork done, and the imaging center. This will help them become familiar so that when the anxiety levels rise, this is something they will not have to worry about.
2. Share your best guesses on the various timelines they will encounter. This includes the initial surgery timeline and the typical NICU stay. Inform them on what dictates some of the lengths of these stays, such as imaging and lab studies. Tell them about what testing will be performed and why. Let them know what follow-up visits will look like and when they will start happening.
3. Make the health system a little less scary and big to them. Describe yourself as an easy point-of-contact for them so they have one number to call.

## Appendix 3. Outline of survey

1. Pre-screening: demographics and did you attend High Risk Pregnancy Counseling (HRPC)?
2. YES for HRPC:
  - Was HRPC at UAB? **(yes/no)**
  - I appreciated having the obstetrician and pediatric surgeon in the same room discussing my pregnancy and the postnatal care for my child.
  - I was more at ease during the pregnancy because of the multiple ultrasounds done prior to the birth of my child.
  - I was well informed and prepared for the delivery of my child.
  - I was well informed and thought the birth plan chosen was best, c-section versus vaginal.
  - I was well informed and prepared for my child’s hospital stay after birth.
  - I was well informed and prepared for the complications that occurred after birth.
  - Was genetic testing utilized? **(yes/no)**
  - I received an accurate picture of what to expect regarding my child’s initial closure procedure.
  - I received an accurate picture of what to expect regarding my child’s initial hospital stay.
  - I felt prepared for the amount/intensity of treatment in the first year.
  - My child has hydrocephalus. **(yes/no)**
    i. My child received one of the following hydrocephalus procedures. **(Shunt or ETV/CPC)**
    ii. My child underwent (please provide a number, e.g. 10) surgical procedures in the first year of life. **(number)**
    iii. This was more or less than I expected. **(much less/less/neutral/more/much more)**
    iv. I received an accurate picture of what to expect regarding my child’s potential to have hydrocephalus.
    v. I received an accurate picture of what to expect regarding my child’s hydrocephalus.
    vi. The reality of hydrocephalus was better than I expected after the prenatal clinic conversation.
    vii. The clinic discussion prepared me for life with hydrocephalus.
  - I received an accurate picture of what to expect regarding my child’s mobility.
  - I received an accurate picture of what to expect regarding my child’s cognitive milestones.
  - I received an accurate picture of what to expect regarding my child’s physical milestones.
  - I received an accurate picture of what to expect regarding my child’s emotional milestones.
  - I received an accurate picture of what to expect in regard to Spina Bifida Clinic.
  - I received an accurate picture of what to expect in regard to my child’s bladder and catheterization.
  - I received an accurate picture of what to expect in regard to my child’s bowel regimen.
  - The Spina Bifida handbooks were useful.
  - I received an accurate picture of what to expect regarding raising a child with spina bifida.
  - I benefited from the discussion in prenatal clinic.
  - What information, if any, did you find to be unhelpful or caused unwarranted anxiety? **(free text)**
  - What information, if any, do you wish would have been described to you? **(free text)**
  - What was the most informative or helpful information you learned? **(free text)**
3. NO for HRPC:
  - I received a prenatal diagnosis. **(yes/no)**
  - I was well informed and prepared for the delivery of my child.
  - I was well informed and prepared for my child’s hospital stay after birth.
  - I was well informed and prepared for the complications that occurred after birth
  - I received an accurate picture of what to expect with my child’s spina bifida diagnosis.
  - I received an accurate picture of what to expect regarding my child’s initial procedure.
  - I received an accurate picture of what to expect regarding my child’s initial hospital stay.
  - Does your child have hydrocephalus? **(yes/no)**
    i. I received an accurate picture of what to expect regarding my child’s hydrocephalus.
  - The discharged teaching I received in the NICU prepared me for taking my baby home from the hospital?
  - I felt prepared for the amount/intensity of treatment in the first year.
  - During your NICU teaching, what information, if any, did you find to be unhelpful or caused unwarranted anxiety? **(free text)**
  - What information, if any, do you wish would have been described to you? **(free text)**
